# Space–time codependence of retinal ganglion cells can be explained by novel and separable components of their receptive fields

**DOI:** 10.14814/phy2.12952

**Published:** 2016-09-07

**Authors:** Cameron S. Cowan, Jasdeep Sabharwal, Samuel M. Wu

**Affiliations:** ^1^ Department of Ophthalmology Baylor College of Medicine Houston Texas; ^2^ Department of Neuroscience Baylor College of Medicine Houston Texas; ^3^ Medical Scientist Training Program Baylor College of Medicine Houston Texas

**Keywords:** Retinal ganglion cells, space–time separability, spatiotemporal tuning

## Abstract

Reverse correlation methods such as spike‐triggered averaging consistently identify the spatial center in the linear receptive fields (RFs) of retinal ganglion cells (GCs). However, the spatial antagonistic surround observed in classical experiments has proven more elusive. Tests for the antagonistic surround have heretofore relied on models that make questionable simplifying assumptions such as space–time separability and radial homogeneity/symmetry. We circumvented these, along with other common assumptions, and observed a linear antagonistic surround in 754 of 805 mouse GCs. By characterizing the RF's space–time structure, we found the overall linear RF's inseparability could be accounted for both by tuning differences between the center and surround and differences within the surround. Finally, we applied this approach to characterize spatial asymmetry in the RF surround. These results shed new light on the spatiotemporal organization of GC linear RFs and highlight a major contributor to its inseparability.

## Introduction

Because of their genetic tractability, mice are an increasingly popular model to study retinal circuitry and its perturbation during disease (Sinclair et al. 2004; Coombs et al. 2006; Abd‐El‐Barr et al. 2009). The output neurons of the mouse retina, ganglion cells (GCs), derive much of their receptive field (light responsivity) from the connection pattern and type of upstream retinal neurons and thus are a readout of retinal function. Understanding how GCs function in mice therefore provides an important platform for investigating retinal function (Wassle 2004; Sanes and Zipursky 2010). A common method for measuring GC function is spike‐triggered averaging (STA) of white noise stimuli, which isolates the linear receptive field (RF) (Meister et al. 1994). This estimate provides a first‐order approximation of the RF which provides insights into the upstream neural circuitry (Field et al. 2007, 2010).

GC RFs are organized into two spatial regions, a center that activates to light onset (ON) or offset (OFF) and an antagonistic polarity surround (Barlow 1953; Kuffler 1953). The center–surround antagonistic RF organization is the basic building block for spatial information processing in the visual system (Hubel and Wiesel 1962; Rodieck 1965; Wu 2010). In linear RFs, the antagonistic surround is present in primates (Chichilnisky and Kalmar 2002; Gauthier et al. 2009) and cats (Reid et al. 1997), and is modeled by a difference of Gaussians (Rodieck 1965). In mice, experiments using spots and annuli or moving gratings found significant surrounds in many GCs (Dedek et al. 2008; Farrow et al. 2013). In contrast, no surround was observed when white noise was used to map mouse linear RFs (Kerschensteiner et al. 2008; Koehler et al. 2011; Della Santina et al. 2013) with the exception of one type of OFF direction‐selective GCs which have both linear receptive fields with achromatic (Kim et al. 2008) and chromatic spatial antagonism (Joesch and Meister 2016). A notable simplification applied by many of these linear RF studies is the difference‐of‐Gaussians model. Reason to question this model has been found in recent research, where local inhomogeneities caused the RF center to differ from a Gaussian profile at high mapping resolutions (Brown et al. 2000; Field et al. 2010; Soo et al. 2011; Schwartz et al. 2012). We therefore hypothesized that similar inhomogeneity in the spatial surround could obscure its presence in the linear RF when the difference‐of‐Gaussians model is strictly applied.

Another factor that may contribute to difficulties characterizing the surround in the GC linear RFs is the way spatial filters are combined with temporal filters into space–time models. GCs of mice and other vertebrates have temporal filters with at least two phases – one fast with center polarity and the other slow and antagonistic (Chichilnisky 2001; Pandarinath et al. 2010; Wang et al. 2011). Such biphasic filters have been successfully combined with spatial Gaussians to describe primate GC responses by relying on an important simplifying assumption: that the cell's spatial and temporal filters are independent of one another (Chichilnisky and Kalmar 2002). Although convenient, this assumption conflicts with observed temporal tuning shifts between the linear RF center and surround (Derrington and Lennie 1982; Enroth‐Cugell et al. 1983; Dawis et al. 1984; Frishman et al. 1987). The presence of center–surround inseparability in GC RFs is therefore acknowledged (Meister and Berry 1999), although questions regarding its strength and form remain.

We recorded a large sample of mouse GCs and confirmed previous reports that, under the assumptions outlined above, the antagonistic surround was weak or absent from their linear RFs. However, when these assumptions were relaxed a strong and pervasive spatial surround was revealed in the same dataset. We therefore systematically assessed the assumptions that underlay the original analysis and discovered significant evidence for space–time inseparability. Importantly, we find that inseparability can be accounted for with a model (termed SoSS) that minimizes complex space–time codependencies. When the SoSS model was applied to our sample of GC linear RFs it revealed five subcomponents with highly distinctive spatiotemporal filtering. Furthermore, the subfilter corresponding to the antagonistic surround was frequently asymmetrically organized supporting its hypothesized non‐Gaussian structure. These results clarify the rules that govern receptive field organization and demonstrate how improved parameterization can reveal its core underlying features.

## Materials and Methods

### Ethical approval

Mice were cared for and handled following approved protocols from the Animal Care and Use Committee of Baylor College of Medicine and in compliance with the National Institutes of Health guidelines for the care and use of experimental animals. All mice were euthanized by cervical dislocation after anesthetizing with isoflurane.

### Electrophysiology

#### Multielectrode recording

Nineteen male C57Bl/6J mice were kept on a regular light/dark cycle and experiments were performed diurnally at 3–4 months of age. Prior to euthanization, mice were dark adapted for at least 90 min. Eyes were removed under infrared illumination using night vision (Nitemare, BE Meyers, Oregon) and their retinas were dissected in a dish containing carboxygenated recording solution. Retinas were placed ganglion cell side up onto nitrocellulose filter paper (0.45 μm HA, Millipore) and transferred onto an electrode array where the preparation was retained with a plastic ring.

The retina was kept at 35.6°C and perfused at 2 mL/min with prewarmed and carboxygenated (95% O_2_, 5% CO_2_) recording medium (in mmol/L: NaCl, 124; KCl, 2.5; CaCl_2_, 2; MgCl_2_, 2; NaH_2_PO_4_, 1.25; NaHCO_3_, 26; and glucose, 22) at pH 7.35 (Tian and Copenhagen 2003). The multielectrode array (MEA‐60, Multichannel Systems, Tübingen Germany) had 60 electrodes spaced 100 μm apart and with diameters of 10 μm. Ganglion cell action potentials were recorded at 20 Khz and prefiltered with a 0.1 Hz high‐pass hardware filter.

### Light calibration

The stimulus was presented from a computer monitor (Dell, SXGA‐JF311‐5100) optically reduced and presented from below the MEA onto the ganglion cell side of the retina. Similar to previous reports (Pandarinath et al. 2010), the ambient light level during an experiment was measured as wavelength‐specific irradiance (*E*(λ), in microwatts cm^−2^) in the plane of the preparation (Thor Labs, S170C and Edmund Optics, SpectraRad). Photon flux in photoisomerizations/photoreceptor/second (*ϕ*) was calculated as $\phi =a_{c}\left(\lambda_{\max}\right)\sum_{\lambda }N_{p}\left(\lambda \right)\tau \left(\lambda \right)S_{r}\left(\lambda \right)$ where $a_{c}\left(\lambda_{\max}\right)$ is the effective collecting area of a photoreceptor at its peak wavelength (0.34 μm^2^ for cones and 0.67 μm^2^ for rods)(Lyubarsky et al. 2004; Pandarinath et al. 2010), $N_{p}\left(\lambda \right)$ is the photon flux per second, and $\tau \left(\lambda \right)$ is wavelength‐dependent transmissivity of the neural retinal (Alpern et al. 1987). Finally, $S_{r}\left(\lambda \right)$ is sensitivity relative to the peak intensity which encompasses the wavelength dependence of both quantum efficiency and molar absorbance coefficients. The ambient light level stimulated rods at 757.9 R*/sec, M‐cones at 384.6 R*/sec, and S‐cones at 8.0 R*/sec.

### White noise receptive field measurements

Receptive fields were mapped for up to 1.5 h using random checkerboards presented at 15 Hz using PsychToolbox (Brainard 1997; Pelli 1997). Each square in the checkerboard was 50 μm on a side and either black or white. Only cells that had a firing rate >0.5 Hz were included for analysis.

### Modeling

#### Center model

To characterize the location and extent of the relatively strong receptive field center, we combined a single two‐dimensional Gaussian with a biphasic temporal filter. The two‐dimensional Gaussian describes the sensitivity *g* at a position (*x,y*) as a function of the location of its center (c_x_,c_y_) and three composite variables a, b, and c.

$$g\left(x,y\right)=k_{1}e^{a{\left(x-c_{x}\right)}^{2}+2b\left(x-c_{x}\right)\left(y-c_{y}\right)+c{\left(y-c_{y}\right)}^{2}}$$

where a, b, and c are dependent on an angle θ and standard deviations in the major σ_x_ and minor axes σ_y_.

$$a=\frac{\cos^{2}\left(\theta \right)}{2\sigma_{x}^{2}}+\frac{\sin^{2}\left(\theta \right)}{2\sigma_{y}^{2}}$$

$$b=\frac{-\sin(2\theta )}{4\sigma_{x}^{2}}+\frac{\sin(2\theta )}{4\sigma_{y}^{2}}$$

$$c=\frac{\sin^{2}\left(\theta \right)}{2\sigma_{x}^{2}}+\frac{\cos^{2}\left(\theta \right)}{2\sigma_{y}^{2}}$$

The temporal filter was the difference in three low‐pass filter impulse responses

$$f\left(t\right)=p_{1}{\left(\frac{t}{\tau_{1}}\right)}^{n_{1}}e^{-n_{1}\left(\frac{t}{\tau_{1}}-1\right)}-p_{1}p_{2}{\left(\frac{t}{\tau_{2}}\right)}^{n_{2}}e^{-n_{2}\left(\frac{t}{\tau_{2}}-1\right)}-p_{1}p_{3}{\left(\frac{t}{\tau_{3}}\right)}^{n_{3}}e^{-n_{3}\left(\frac{t}{\tau_{3}}-1\right)}$$

where *f*(*t*) is the filter strength in time bin *t* before the spike, and *p*, τ, and *n* are all fit parameters that shape the temporal filter.

These two filters are combined and regressed against the raw STA ω.

$$\omega \left(x,y,t\right)=g\left(x,y\right)f\left(t\right)$$

#### Spatial pooling and temporal characterization

The spatiotemporal fit of the receptive field center was used to divide the spatial inputs into up to nine annular regions, each spanning one standard deviation (SD) of radial distance. Temporal STAs within these annuli were pooled by either summing or averaging, which generally improved signal‐to‐noise ratios. Initially this temporal filter was fit on a per‐annulus basis using a standard least squares regression. Appropriate temporal models were determined by starting with a simple line at zero and iteratively adding additional temporal subfilters, statistical improvement was assessed with an F‐test. Each temporal subfilter was an impulse response of a low‐pass filter as shown in Equation (2). Subfilters were tracked across annuli by clustering the subfilter population using a three‐dimensional mixture of Gaussians model fit on p, τ, and n where the number of clusters was determined by an F‐test.

#### Assessing space–time separability

Singular value decomposition (SVD) was applied on the STAs that had been transformed into two‐dimensional space (radial distance and time). SVD creates a set of separable subfilters, ordered by decreasing power, and has therefore found use as a test for space–time separability in the visual (Mazer et al. 2002) and auditory systems (Depireux et al. 2001).

#### The sum of separable subfilters (SoSS) model

The SoSS model combines up to five subfilters, each with a unique temporal and spatial component (see eq. (1)). Each subfilter's temporal properties were described by a low‐pass filter impulse response and its spatial extent was described by a two‐dimensional Gaussian. All subfilters for a cell had the same spatial center and rotation, and their temporal properties were constrained to match the five groups identified by clustering in Figure 3. Excellent results were achieved in subsequent experiments when subfilter parameters were initialized to their expected values and constrained only for sanity.

To perform a regression on the annular‐averaged data, we first evaluated the model at the center of each spatial input whose center lay within a given annulus, and then averaged these predictions to achieve an annulus prediction. Second, because the number of spatial inputs varied between annuli, the error terms used in a standard regression would be inconsistent across annuli. We corrected for this by using a weighted least squares regression, where the weights are the square root of the number of spatial inputs in each annulus. This factor is derived from the central limit theorem, and acts to level the effect of noise across annuli.

#### Identification and assessment of receptive field hotspots

For each cell that had one of the two surround‐associated subfilter types (4 and 5), we compared the temporal STA of each spatial input to the model prediction for that input (henceforth referred to as the probe) by calculating their zero‐lag cross‐correlation (x‐corr). These x‐corr values vary in magnitude based on the strength of the signal, the noise, and probe. To estimate the significance of the probe‐to‐signal interaction, we made a reference comparison of the probe to a simulated population of noise STAs. Our stimulus was binary, so noise STAs were generated by averaging a number of 1s and ‐1s equivalent to the number of spikes averaged in the original STA. The standard deviation of the x‐corrs from the probe to simulated noise comparison was used to normalize the probe to STA comparison. Any inputs that had a normalized STA to probe x‐corr of greater than 3 SD were classified as surround hotspots.

The ability of the hotspots to capture the temporal signal present in surround was estimated by linear regression of the β between the summed temporal STAs in the hotspots *h* and in the full surround *s*

s=βh

To determine the strength and orientation of asymmetry, we rotated each cell so that its major axis aligned with the *x*‐axis and then normalized the major and minor axis by their respective sizes. We then calculated a center of mass for the set of hotspots. This gives a larger weight to distant inputs, but ignores the relative strength of the hotspots. A cell was designated as asymmetric if the distribution of its hotspot distances was significantly different from the centered origin as determined by a Wilcoxon signed‐rank test.

### Statistical tests

Specific tests used and *P*‐values are indicated in the text and methods. For model comparisons we used an *F*‐test to compare a constrained and unconstrained model. For comparison of populations we used the Student's t‐test when normally distributed, otherwise we used a Wilcoxon signed‐rank test or Mann–Whitney *U* test. In all cases we applied a Bonferroni correction to account for multiple comparisons.

## Results

**Figure 1:**
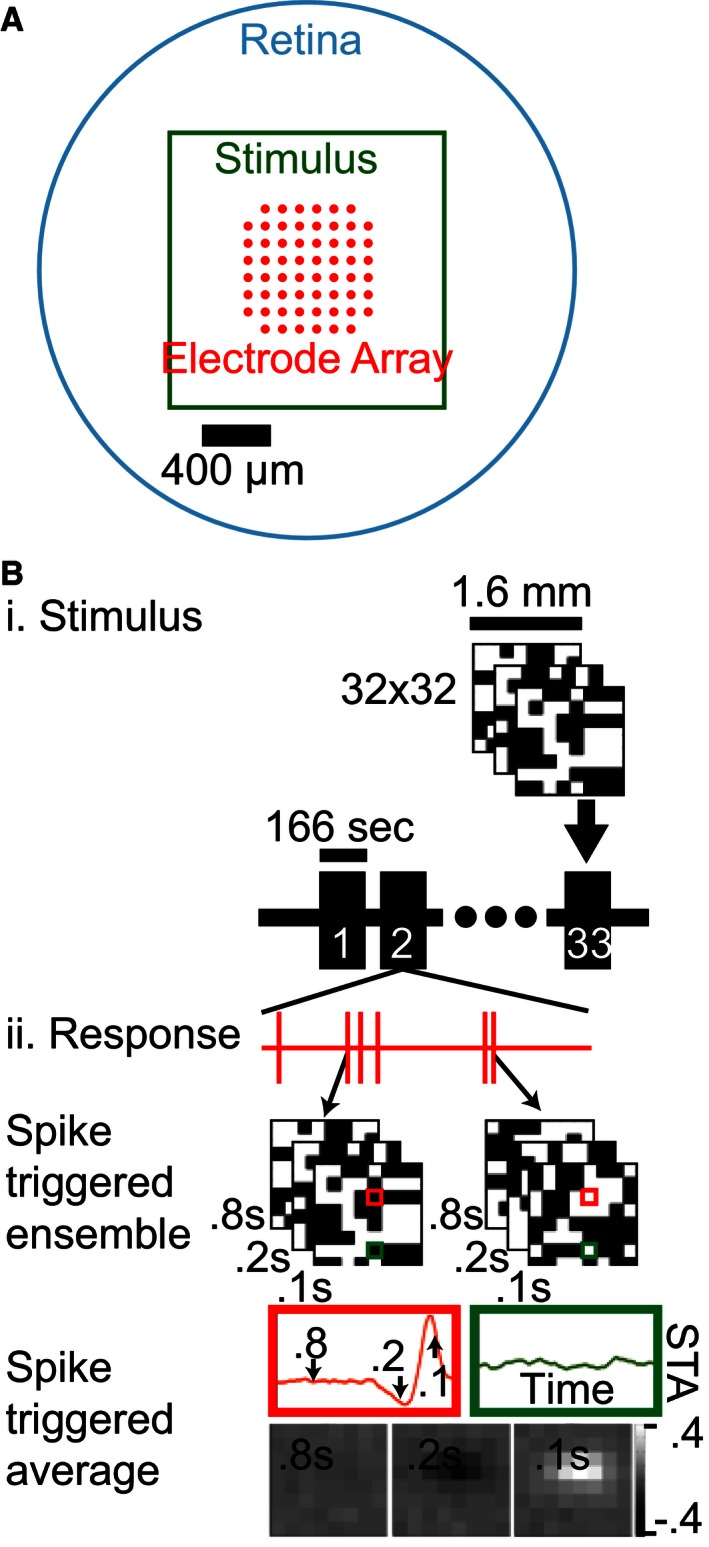
Recording and stimulation of ganglion cells. (A) An explanted mouse retina (cyan) was placed on a multielectrode array (red) ganglion cell side down and stimulated with an optically reduced monitor image (green). (B) i: The stimulus was an iterated sequence of 32 × 32 binary white noise checkerboards (8 × 8 illustrated). ii: Spiking responses of individual cells were identified and used to calculate the average stimulus that triggered a spiking response, the spike‐triggered average (STA). The resulting receptive field map had a space–time structure which can be illustrated as a temporal filter for each spatial point (red and green highlights) or as a spatial filter at a single temporal slice (bottom).

We used an electrode array to record ganglion cells (GCs) from flat‐mount mouse retinas during visual stimulation, as illustrated in Figure 1A. Stimuli consisted of white noise checkerboards displayed in series which were used to generate spike‐triggered averages (STAs) for GCs, as illustrated in Figure 1B. Checkerboards were 32 × 32 grids of black or white 50 μm squares and were presented at 15 Hz for a total of 166 sec per trial. The mean light level activated rods at 757.9 R*/sec, M‐cones at 384.6 R*/sec, and S‐cones at 8.0 R*/sec. These parameters were chosen to maximally drive GCs, thereby improving our ability to detect weak signals in the receptive field surround. Example results from this process are illustrated in Figure 1Bii; temporal STAs are shown at two spatial inputs (next to bottom row) and spatial STAs are shown at three temporal slices (bottom row).

### Linear receptive fields of mouse GCs have an antagonistic surround

While previous studies in mice have observed antagonistic surrounds in response to stimuli with strong space–time correlations (Dedek et al. 2008), no surround was observed when linear receptive fields were mapped with white noise checkerboards (Kerschensteiner et al. 2008; Koehler et al. 2011; Della Santina et al. 2013). Analyses in these studies relied on the difference‐of‐Gaussians model which may obscure the surround if it is not Gaussian distributed with radial homogeneity/symmetry. We therefore launched a systematic reexamination of the linear receptive field by replacing this restrictive assumption with a more general approach.

**Figure 2:**
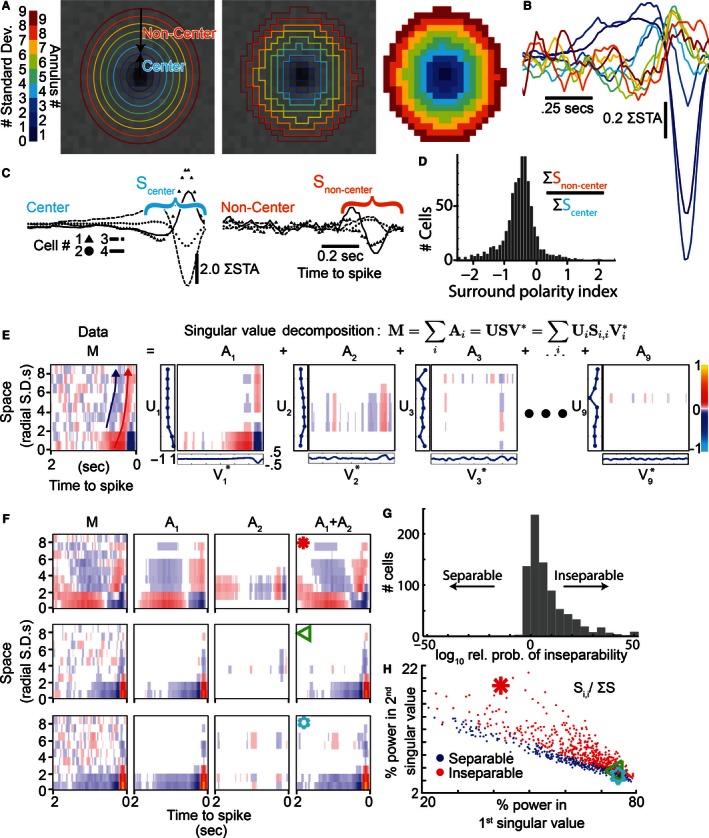
Ganglion cells (GC) linear receptive fields have antagonistic surrounds and many are space–time inseparable. (**A**) A single Gaussian profile was fit in space–time to localize the receptive field center. Its radial distance isoclines are shown in color for 1σ intervals (left). These annuli are then used to group the spatial inputs into up to nine annular regions (middle and right). (**B**) Temporal STAs were summated within the annular regions to help determine their dependence on radial distance. (**C**) To test for a surround, inputs were further grouped into the receptive field center (≤3σ, cyan) and noncenter regions (>3σ, orange) which is illustrated for four example cells. (D) A histogram comparing the first 0.33 seconds of the center (S_center_) and noncenter (S_non‐center_) responses from all cells, as shown in (C). The predominance of values below zero reveals an antagonistic surround was pervasive in the population. (E) A space–time receptive field map (M, left) that replots the information from (B) into an image using a color map (far right). Singular value decomposition divides the raw STA (M) into nine separable space (U, traces to left of images) and time filters (V, traces below images) that are combined to create the matrices A_1_ to A_9_. This cell's space–time inseparability can be seen in the spatiotemporal codependence exhibited by the diagonal drift in its raw STA (red and blue arrows) and by the significant power/structure of A_2_. (F) The receptive field map (M), first two singular matrices (A_1_, A_2_), and their sum (A_1_+A_2_) are shown for three example cells; the top GC (red star) was strongly inseparable, the middle (green triangle) was still inseparable, and the bottom (cyan star) was separable. (G) The relative likelihood of space–time inseparability was higher for the majority of GCs (corrected Akaike information criterion between the A_1_ and A_1_+A_2_ models). (H) Space–time inseparable cells (red, probability of separability < 0.01 from G) had high power in their second singular value compared to the first. Space–time separable cells (blue) had a lower relative power in their second singular value.

The structure of the receptive field center was determined by fitting the product of a single 2D spatial Gaussian and a triphasic temporal filter to each cell's spatiotemporal STA. These parameters were used to delineate a set of isoclines at 1*σ* intervals (Fig. 2A, left) which were used to group each spatial input into an annular region (middle and right). The inputs within the inner 3*σ* were collectively termed the receptive field center as they contained the majority of its signal, and the inputs from 3*σ* to 9*σ* were termed the noncenter receptive field. Figure 2B shows the dependence of the summated temporal STAs on radial distance, colored to match the annular distance from Figure 2A. While this regularization is not lossless, applying it to the checkerboard‐derived data allows us to characterize the spatiotemporal dependence of many cells’ noncenter receptive fields simultaneously without assuming radial homogeneity or symmetry. The central spatial inputs of this cell (blue tinted traces, Fig. 2B) have a strong preference for light offset (negative deflection), whereas the noncenter regions have a preference for light onset that is consistent with an antagonistic surround.

To assess the prevalence of the linear antagonistic surround, we first summated the temporal STAs of the spatial inputs from the noncenter region. Figure 2C shows this analysis for four GCs (marker shapes), each had a summed temporal STA in the noncenter region (right) with a polarity antagonistic to the center (left). We quantitatively assessed the surround by calculating the ratio of the center and noncenter signals in the first 333 ms (S_center_ and S_non‐center_ in Fig. 2C). The population data show that the surround polarity index was less than zero in the majority of cells (88.7%), indicating the linear antagonistic surround was pervasive (Fig. 2D, *n* = 805 cells). To show that this result benefits from our more general approach we applied the difference‐of‐Gaussians fit to the spatial profile of the space–time receptive field, and only observed a linear surround in 29.8% of GCs.

### GC linear receptive fields exhibit varying degrees of space–time inseparability

As a surround was observed in the majority of GCs, we proceeded to study its spatiotemporal properties. This analysis was carried out using the nine spatially collapsed temporal STAs shown in Figure 2B. These spatiotemporal profiles provide an excellent opportunity to determine whether the center and surround are space–time separable. Separable systems can be factored into a product of spatial and temporal filters, whereas this is not possible in inseparable systems as the temporal filter can vary with space or vice versa. Despite the role of space–time inseparability in encoding complex visual stimuli (Meister and Berry 1999) it is often ignored in linear RF analyses (Chichilnisky and Kalmar 2002). In the leftmost panel of Figure 2E, the STA from Figure 2B is projected in two dimensions, time and radial distance, with filter strength and polarity represented by color. The apparent shift in temporal tuning with space (Fig. 2E, arrows) is suggestive of inseparability. To quantitatively assess separability in the time‐radial distance domain we used SVD, a standard technique that breaks down data into separable components as shown in equation (1) (Depireux et al. 2001; Mazer et al. 2002), where **M** is the data matrix, **U** is a spatial filter matrix in the radial distance domain, **S** is a diagonal matrix of weights, **V** is a temporal filter matrix, *i* is the index of the singular value, and ***A***_***i***_ is the space–time filter for *i*th singular value.

(1) $$\omega \left(r,t\right)=M=\sum_{i}A_{i}={\mathrm{USV}}^{\;*}=\sum_{i}U_{i}S_{i,i}V_{i}^{\;*}=\sum_{i}g_{i}\left(r\right)f_{i}\left(t\right)$$

Figure 2E shows the decomposition of an example cell's spatiotemporal STA (ω or M) into a set of matrices (A_1‐9_), which contain the outer product of a spatial filter (U_i_, vertical axis) and a temporal filter (V_i_, horizontal axis), scaled by the *i*th singular value (S_i,i_). A_1_–A_9_ are ordered by descending power and represent separable space–time components of the original receptive field (M). The rightmost segment of the equation is a parameterization of the spatiotemporal dependence, which will be discussed subsequently. Characteristic of inseparability, the signal‐derived variation in M does not appear to be captured by a single separable space–time filter (A_1_). Rather, significant power and structure is present in the second filter (A_2_).

Using this approach, we characterized the degree of space–time inseparability in a population of retinal GCs (*n* = 805). Three examples are shown to highlight the variation in the population in Figure 2F: one highly inseparable (top row, red asterisk), one borderline inseparable (middle row, green triangle), and one separable (bottom row, blue star). In the inseparable examples A_1_ + A_2_ (fourth column) faithfully reflects the space–time structure of M, whereas in the separable cell A_1_ alone (second column) is sufficient.

The above represents a qualitative description of inseparability, to quantify it across the population we used the Akaike information criterion to compare a model including only the weights from the first singular value (representing separability) to a model including the first and second singular values (representing inseparability). The histogram in Figure 2G shows the distribution of the relative probabilities of inseparability for all reported ganglion cells (*n* = 805). A threshold value of 100 (meaning the inseparable model was 100 times more likely to be needed) was used to identify inseparability. While some mouse retinal GCs were approximately space–time separable, a substantial number were inseparable (24.1%). Separable cells (Fig. 2H, blue points) tended to have a lower percent power in the second singular value relative to the first, whereas inseparable cells (red points) had a relatively higher percent power in the second singular value. The three sample cells are indicated by the matched symbols. We conclude that a general model of GC center–surround linear receptive fields must not assume space–time separability.

### Receptive fields can be decomposed into five space–time separable subfilters

We sought to identify a model that accounts for the change in temporal filtering with space observed in Figure 2. Because multiple component phases combine to give GCs their band‐pass characteristic (Chichilnisky and Kalmar 2002), we quantified the properties of each average STA by modeling it as a summed set of temporal subfilters. This model is shown in equation (2), where each subfilter is a low‐pass filter impulse response *f* indexed by *i* and parameterized by magnitude *p*, time‐to‐peak *τ*, and the number of stages in the filter *n* (Chichilnisky and Kalmar 2002). By tracking the spatial dependence of these temporal parameters, we can study whether/how each subfilters’ space–time dependence contributes to inseparability in the overall temporal filter.

(2) $$f_{i}\left(t\right)=\frac{p_{i^{\;*}}{\left(\frac{t}{\tau_{i}}\right)}^{n_{i}}\times e^{-n_{i_{}^{\;*}}\left(\frac{t}{\tau_{i}}-1\right)}}{e^{-n_{i}-1}}$$

**Figure 3:**
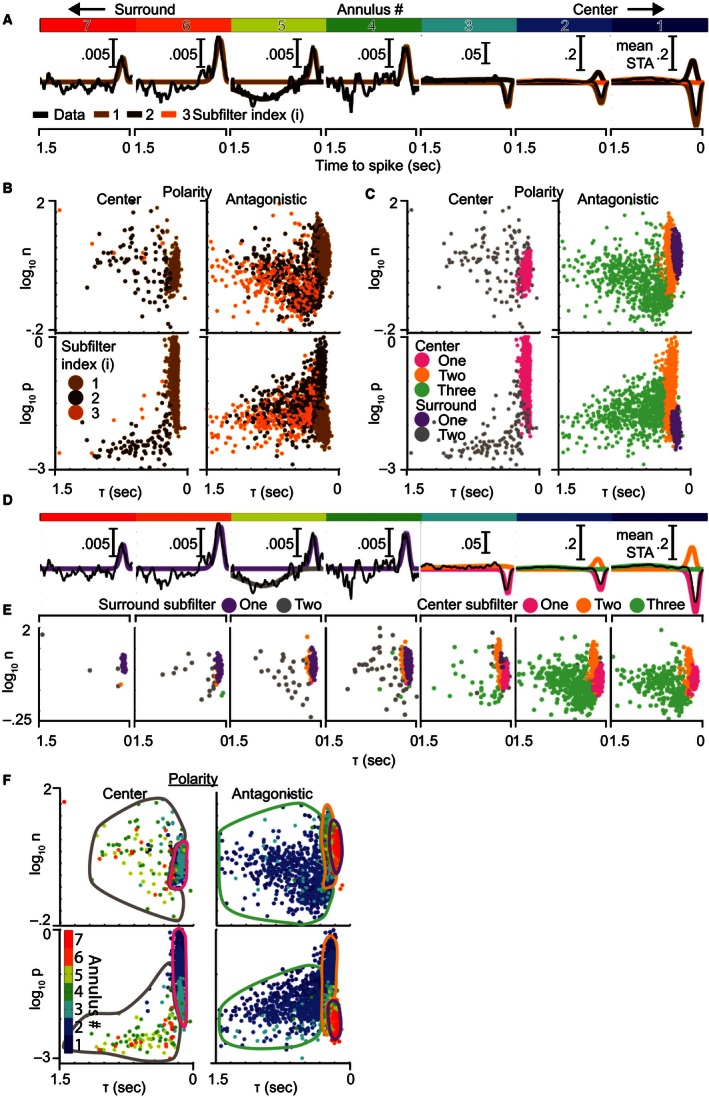
Ganglion cell temporal STAs are composed of subfilters with five distinct patterns of spatiotemporal tuning. (A) The average temporal STAs (black traces) within annuli at different radial distances from the receptive field center (see Fig. 2) were each fit by the sum of up to three subfilters (brown/orange traces). Each subfilter is the impulse response of a low‐pass temporal filter. Annular distance is indicated by the primary‐colored bar at the top, with central annuli on the right. (B) Comparison of subfilter properties from all annuli: amplitude (|p|), delay to peak (*τ*), and filter order (*n*). Subfilters are divided into center and antagonistic groups based on their polarity relative to the receptive field center. Colors are carried from (A) to illustrate how the subfilter population is obtained. (C) The same plot, recolored into five subfilter types (pastel colors) based on a three‐dimensional (p, τ, n) mixture of Gaussians clustering. Clusters were identified as center or surround based on D and E. (D) The same cell from (A) is shown with its component subfilters colored by the types from (C). Center subfilter types 1 (magenta), 2 (orange), and 3 (green) are located in the central annuli, whereas surround subfilter types 1 (purple) and 2 (gray) are in more distant annuli. (E) The dependence of subfilter type on radial distance is illustrated by breaking plot (C) down by radial distance and combining both polarities. (F) The subfilters from (C) have been recolored by their radial distance from center to demonstrate the dependence of subfilter properties on radial distance. Bordered regions have been added to approximate the boundaries between the subfilter types shown in (C). Within each subfilter type, there is a strong dependence of p on radial distance (vertical rainbow effect in the bottom plots), but no obvious codependence for τ or n. This indicates that individual subfilter types vary their scale but maintain their shape over space, suggesting they are space–time separable.

Figure 3A shows an example fit of the average STAs (black lines) at different annuli (indicated by the segments in the top color bar). Whereas the model is fit to the data with the subfilters summed, Figure 3A illustrates the individual subfilters (RGB colored lines). To estimate the number of subfilters at each annulus, we initially assumed a null model and iteratively added subfilters until no longer statistically justified by an *F*‐test (*P* < 0.01). For the illustrated cell, the first subfilter (light brown line) was observed in all annuli, whereas a second (dark brown line) and third subfilter (orange line) were identified more frequently in the receptive field center (annuli 1–3). A fourth subfilter, observed at only chance rates across the population, was excluded. Notably, this is the first evidence for a three‐subfilter model in the receptive field center, which confers dual band‐pass frequency filtering (not illustrated). In total, this cell would have 13 subfilters. To determine if 13 distinct subfilters are indeed needed, as compared to a combination of fewer subfilters that change their size across space, we look at these subfilters across the population.

The subfilters from these fits are shown for a population of GCs (*n* = 778, cells with model fits) in Figure 3B. Each point in the scatter plots represents one subfilter from one cell's STA at one annulus. Subfilters were divided into center (left column) and antagonistic polarity groups (right column), and each was plotted according to its τ and n (top row), and τ and p (bottom row). Figures 3A and B are color matched by subfilter index to illustrate the subfilter identification process, but this indexing was not used in subsequent analyses. Instead, groups of subfilters were clustered based on the similarity of their temporal properties (Fig. 3C). We hypothesized that these groups contain subfilters of the same type, but at different annuli. If true, the temporal properties of subfilter types could be tracked across space, furthering our goal of understanding the shifts in the overall temporal filter. Clustering revealed five subfilter types. The response polarity of types 1 (magenta) and 5 (gray) matched the receptive field center, whereas types 2 (orange), 3 (green), and 4 (purple) were antagonistic.

The same example cell from Figure 3A is recolored in Figure 3D to indicate the type of each subfilter. Rather than using 13 distinct subfilters, only 5 subfilters with distinct properties were needed to explain the cell's annular STA. In the receptive field's spatial center (annuli 1‐3), only subfilter types 1, 2, and 3 were present so they will henceforth be referred to as center subfilters 1, 2, and 3. On the other hand, subfilter types 4 and 5 were observed in the surround and will henceforth be termed surround subfilters 1 and 2. This is just a sample cell, and these five subfilters would work similarly for almost all cells in our population. The population data in Figure 3E demonstrate the same enrichment of these subfilters in the center and surround. Consistent with our hypothesis, each subfilter type was present over a range of annuli allowing us to track the properties of each subfilter type over space.

Figure 3F recolors Figure 3C to indicate the radial distance (dot color) of each subfilter (dots) and encircles the regions to indicate the subfilter types from Figure 3C (colored borders). If subfilter types (regions) have temporal properties (position) that are dependent on radial distance (color), then the color within each cluster will drift with radial distance. The top two plots do not exhibit color drift within subtypes indicating that the properties describing temporal filtering, time‐to‐peak (*τ*,* x*‐axis), and filter order (*n*,* y*‐axis) are not dependent on radial distance. Conversely, subtypes in the bottom two plots showed vertical color progression (darker blue above lighter blue) signifying decreasing response magnitude (p, *y*‐axis) with increasing radial distance. In summary, the shape of the subfilters’ temporal profiles, as determined by τ and n, does not vary meaningfully with radial distance but their scale (magnitude) does. We therefore conclude that space–time separability can be applied to individual subfilters which are then combined to form an inseparable overall filter.

### Mixing space–time separable subfilters accounts for GC center–surround responses

**Figure 4:**
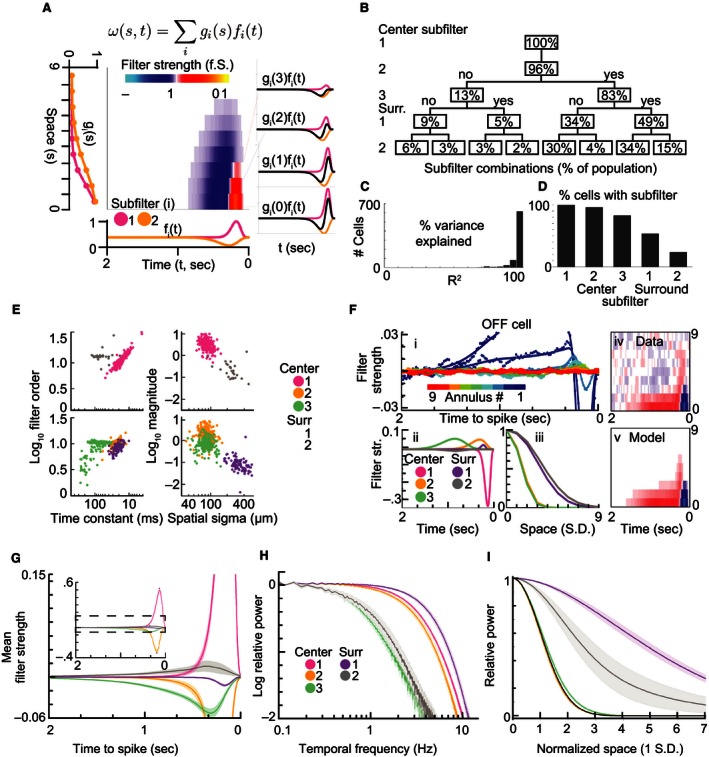
A model for ganglion cell receptive fields consisting of multiple separable subfilters. (A) The outer products of two pairs (orange and magenta) of spatial g_i_(s) and temporal subfilters f_i_(t) are summated to generate a space–time inseparable receptive field map. As shown at right, at a given radial distance (s) from the center, the model's temporal filter is the spatially weighted sum (black trace) of the first (magenta trace) and second (orange trace) subfilters. (B) A sequence of F‐tests were used to determine the number of subfilters that were statistically justifiable (*P* < 0.01 after post hoc correction), the percent of cells reaching each level is visualized. (C) The preferred model explained the majority of the variance in the space–time receptive field. (D) Center subfilters 1, 2, and 3 were present in the majority of cells, whereas surround subfilters 1 and 2 were observed less frequently. (E) i: Models fits (traces) to annular‐averaged STAs (dots) for an example OFF (left) cell. The paired temporal (ii) and spatial (iii) components of the individual subfilters. The space–time codependence in the receptive field map (iv) is accounted for by the model (v). (F) The spatiotemporal properties of the observed subfilters fell into five distinct clusters. Filter time constants are compared to filter order for center polarity (i) and opposite polarity subfilters (ii). Spatial extents are compared to filter magnitude for center polarity (iii) and opposite polarity subfilters (iv). (G) Population average temporal filters from each subfilter type. (H) The same temporal filters averaged in the frequency domain. (I) The average spatial filters for each subtype showed no difference between center subfilters 1, 2, and 3, but surround subfilters 1 and 2 were each significantly different. The first subtype was constrained to a unit normal distribution, and is therefore illustrated in black. Shaded regions in all plots are ±3 SE.

Based on the finding that the receptive field can be described by combining subfilters that are individually space–time separable, we employed a model similar to the result of the SVD, but where the separable spatial and temporal components were replaced by temporal f(t) and spatial g(s) functions (eq. (1)). In contrast to the model applied in Figure 3, this constrains each subfilter's temporal properties to retain their shape across annuli and be scaled according to a Gaussian spatial profile. This model is illustrated in Figure 4A, where the overall linear filter ω is composed of *i* subfilters, each with a dependence on radial distance *r* that is represented by a peak‐normalized Gaussian distribution *g(s)* and its dependence on time *t* is expressed by its temporal profile *f(t)* (Equation (2)). The interactions between two artificial spatial and temporal profiles are illustrated in Figure 4A. As a consequence of their different spatial profiles, the relative weights of the subfilters vary with space causing the temporal tuning to drift (diagonal white arrow). This illustrates how separable subfilters can be combined within the model to generate an inseparable overall filter.

We used this model to examine 805 mouse GCs across 19 retinas. Up to five subfilters were included, each constrained to match the temporal profiles and spatial extents of the corresponding subfilter types from Figure 3 (see methods). As it remains uncertain whether all cells contain the five subfilter types, we adopted an iterative procedure, similar to that in Figure 3B, to estimate the distribution of subfilter combinations in mouse GCs (Fig. 4B). We first compared a model containing only subfilter type 1 to a null model, and found significant evidence for its inclusion in 100% of GCs (*F*‐test at *P* < 0.01, post hoc corrected). We added subfilter type 2 to the preferred model from the first step and found its inclusion was justified in 96% of GCs. In subsequent steps, center 3, surround 1, and surround 2 were iteratively added to the preferred model to generate the distribution of subfilter combinations shown in Figure 4B. For example, the bottom right node shows that 15% GCs had all five subfilter types, whereas the bottom left node shows that 6% had only centers 1 and 2.

To determine if the model accurately describes the signal in the STA, we evaluated the goodness of fit of the preferred model from Figure 4B for each cell. Figure 4C shows that more than 95% of the variance in the data was explained by the model in 634 of 805 GCs. The percentage of cells observed with each subfilter type, derived from Figure 4B, is shown in the bar graph in Figure 4D. There was a progressive decline in observation frequency with increasing subfilter type, bottoming at 24% (3 + 2 + 4 + 15%) for surround 2. Based on the results from Figure 4B–D, the uniform application of the full five subfilter model may not be necessary. On the contrary, at least three subfilters were present in 91% of GCs.

The model fits from two example cells are shown in Figure 4E–F. For both cells the top left panel (i) shows the annulus‐averaged STA (dots) and its corresponding model fit (lines). Below, the temporal (panel ii) and spatial (panel iii) profiles are shown for each subfilter type (pastel colors). To highlight any drift in their spatiotemporal dependence, the data and model from panel (i) are replotted as spatiotemporal maps in panels (iv) and (v). The OFF‐dominated cell in Figure 4E preferred the inclusion of all five subfilter types in the model, whereas the ON‐dominated cell included only four. The space–time inseparability of these cells manifests as the progressive shift of the slow antagonistic component in the center (panel iv, red region in the bottom middle) to earlier times in the surround (toward the top right). The model appeared to faithfully capture this inseparability (panel v).

Figure 4G shows the population average temporal profiles (lines, shaded regions are 3 SE) for the five subfilter types (colors). Positive/negative values on this *Y*‐axis represent center/surround polarity rather than the ON/OFF polarity used in Figures Eii and Fii. Figure 4H shows the same information as Figure 4G in the frequency domain, generated by averaging the normalized power spectral density functions of individual GCs for each subfilter type. Lastly, the average spatial profile of each subfilter type is shown in Figure 4I. These results indicate center 3 and surround 2 have lower temporal frequency tuning (3 dB attenuation near 0.8 Hz), whereas centers 1 and 2 and surround 1 are tuned to higher frequencies (3 dB attenuation near 5 Hz). In addition, the three subfilters located in the spatial center had indistinguishable spatial extents, whereas surround subfilter 1 extended further into the surround than surround subfilter 2.

### ON‐ and OFF‐dominated GCs have subfilters with different spatial and temporal properties

**Figure 5:**
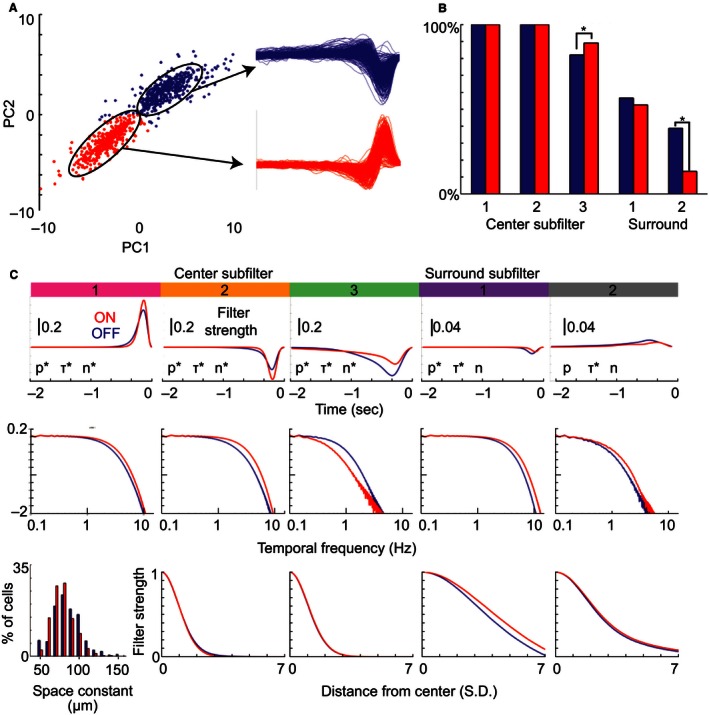
Subfilter properties differ significantly between ON‐ and OFF‐dominated cells. (A) Cells are plotted relative to the first (PC1) and second principal components (PC2) of the temporal STAs at their spatial peak. Two clusters were apparent, the blue group corresponded to OFF‐dominated cells and the red to ON‐dominated cells. (B) Comparison of subfilter observation rates between OFF‐ (blue) and ON‐dominated cells (red). (C) For each subfilter type (columns) we compare the temporal impulse responses (top row), frequency tuning (middle row), and spatial profiles (bottom row) of ON‐ and OFF‐dominated cells. As the first subfilter was constrained to match the original temporal fit, its spatial profile (bottom left) is substituted with the distribution of radial space constants. The asterisk next to p, *τ*, and *n* indicates those properties differed significantly between ON‐ and OFF‐dominated cells (*P* < 0.01, *t*‐test with post hoc correction).

As GC populations recorded on the MEA contain subtypes with different morphological and physiological properties (Wassle and Boycott 1991; Masland 2001), we tested whether subfilter properties varied between subpopulations consisting of different subtypes. In particular, we asked whether ON‐ and OFF‐dominated GCs varied in their spatiotemporal tuning, as has been observed previously (Zaghloul et al. 2003; Murphy and Rieke 2006; Pandarinath et al. 2010), and whether these shifts differed across subfilters. GC subtypes were discriminated by performing principal component (PC) analysis on normalized temporal STAs (see methods) from a population of GCs (Field et al. 2007). The scatter plot in Figure 5A compares PC1 and PC2 from this analysis, and shows two distinct clusters. These clusters had temporal STAs that corresponded to OFF‐ (blue) and ON‐dominated (red) GCs. We did not further classify cells based on response to full field light or directional gratings.

As GCs of the same subtype have receptive fields that tile the receptive field with minimal overlap (Devries and Baylor 1997), we probed the subtype composition of the clusters by measuring their GCs’ overlap. The internal overlap in the ON‐ and OFF‐dominated GCs was 8.1% and 6.8%, respectively (not shown), consistent with the expectation that each cluster is composed of multiple subtypes.

Figure 5B shows the relative frequency of the five subfilter types in ON‐ and OFF‐dominated GCs. In the receptive field center, centers 1 and 2 were always present in both ON‐ and OFF‐dominated GCs but center 3 was more frequently observed in ON‐dominated cells (*P* < 0.01). Among the surround‐associated subfilters, surround 1 was observed in 52.6% of ON‐dominated and 56.6% of OFF‐dominated GCs (n.s.), but surround 2 was observed in 38.8% of OFF‐dominated GCs but only 13.3% of ON GCs (*P* < 0.01).

Figure 5C compares the average spatial and temporal properties of each subfilter type in ON‐ (red) and OFF‐dominated GCs (blue). The first row compares the temporal impulse responses, the second their normalized frequency filtering, and the third their normalized spatial profiles. Each column contains one subfilter type, ordered from center 1 (leftmost) to surround 2 (rightmost). The temporal profiles of all five subfilters types differed statistically between ON‐ and OFF‐dominated GCs (*P* < 0.01) based on a post hoc corrected rank sum test on the parameters underlying the temporal model (Fig. 5C, significance of each parameter – p, *τ*, and n – indicated by an asterisk). The temporal filters of ON‐dominated cells were stronger than OFF‐dominated cells in centers 1 and 2, but weaker in center 3 and surround 1. Center 1/2 and surround 1/2 were tuned to higher frequencies in ON‐dominated cells, but center 3 had significantly higher tuning in OFF‐dominated cells. The normalized spatial profiles (third row) were statistically indistinguishable for all but surround 1, which was significantly wider in ON‐ than in OFF‐dominated GCs. If these subfilters were all driven by the same source we would not expect to see differences between ON‐ and OFF‐dominated groups (center subfilters 1–3 should all shift in the same direction). The differential shifts in specific subfilter types provide evidence that subfilter types are driven by distinct underlying processes.

### Identification of hotspots in the GC antagonistic surround

**Figure 6:**
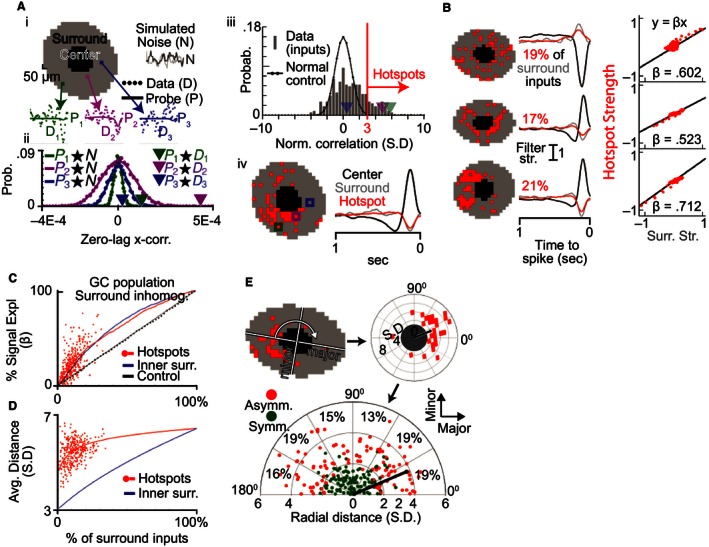
The receptive field surround contains spatially nonuniform hotspots. (A)i: Raw STA data points (D) are shown for three example inputs in one cell's spatial surround (gray area). The trace through the points is generated using the model fit to the average data (see Fig. 4) and is used as a probe (P) for surround signal in each input. As a control, probes are also compared to simulated STAs that contain only noise (N). Aii: Zero‐lag cross‐correlation is used to compare each probe with the simulated noise, yielding a normal probability distribution unique to each probe (dots and traced distribution) which is used to assess the significance of the probe's correlation with the STA data (arrowheads). Aiii: The data–probe comparisons from (ii) were normalized by the variance of the noise–probe comparisons and the normalized correlations for one cell are binned into this histogram. The observed correlations are positively skewed relative to the unit normal distribution (black trace) that would be expected in the absence of surround signal. Inputs were defined as surround “hotspots” if their normalized cross‐correlation exceeded 3 SD (red vertical line). Aiv: The spatial distribution of surround hotspots (left) and the summed temporal STA in the center (black trace), hotspots (red trace), and surround including hotspots (gray). (B) Example cells with hotspots that were diffusely (top) or densely distributed (middle and bottom) and symmetrical (top and middle) or asymmetrical (bottom). The amount of signal in the surround's summed temporal STA (middle) was estimated by calculating the β between the hotspots and the overall surround strength (right). (C) As a function of the percent inputs in the surround, β between hotspots and the surround was high across the GC population (red dots). The hotspot approach (red trace) outperforms both a random control method (black) and was comparable to selecting the innermost surround inputs first (blue trace). (D) Inputs selected by the hotspot method (red dots) were significantly more distant than when choosing the innermost surround inputs first (blue). (E) Surround asymmetry relative to the major and minor axes of the elliptical receptive field center (top left) was assessed by rotating and normalizing the spatial field of hotspots (top right). The center of mass (black dot and line) of the hotspot inputs (red) is illustrated for an example cell. The bottom polar plot shows that the cell population (*n* = 256 cells with >=15 hotspot inputs) often had strongly asymmetrical hotspots (red points, sign‐rank test). There was no significant statistical bias in the major or minor axis distribution of the asymmetry. (*χ* ^2^ test).

Because inhomogeneity has been observed in the receptive field center at high mapping resolutions (Soo et al. 2011), we next evaluated whether the spatial surround's microstructure exhibits a similar departure from a Gaussian profile. We approached this question by applying the model derived in the previous sections to predict the STA signal for checkerboard inputs at different radial distances in the surround (see methods). For every checkerboard input this gave us a model prediction (probe) and raw STA (data). We used zero‐lag cross‐correlation (x‐corr) to compare the probe to the data within each spatial input (derived from one checkerboard square). Figure 6 Ai shows data (dots, D) and probes (lines, P) from three different spatial inputs (colors) and Figure 6 Aii shows their corresponding data/probe x‐corr values (arrowheads). The data and probe of input 2 (purple) match closely, resulting in a large x‐corr value, whereas the data of inputs 1 (green) and 3 (blue) appeared to be larger and smaller, respectively, than their corresponding probes. To provide a frame of reference for each x‐corr value, we generated a control population (Figure 6 Aii, dots) by calculating the x‐corr between each probe and a population of simulated STAs containing no signal (N, or noise). Each control population had a normal distribution with an expected mean of zero and a characteristic variance (Fig. 6 Aii, lines). We normalized all data/probe x‐corr values (arrowheads) by dividing by the standard deviation of the control noise population. As outlined in subsequent sections, cross‐validation was employed at each step to provide an unbiased evaluation of this approach.

### Hotspots efficiently account for surround signal and have a non‐Gaussian distribution

Figure 6Aiii shows the result of this normalization for the full population of surround inputs which have been binned into a probability histogram (gray bars), and highlights the same three example inputs (arrowheads). If only noise was present in the surround, the histogram in Figure 6Aiii should follow the unit normal distribution indicated by the black line. Instead, we found the histogram was positively skewed indicating the widespread presence of signal matching the probe. To determine the spatial organization of this signal in the surround, we defined all spatial inputs with an x‐corr greater than 3*σ* (red line) as “hotspots”. The left side of Figure 6Aiv shows that the spatial distribution of hotspots in this cell was asymmetrical and the colored boxes highlight the locations of hotspot inputs 1 (green) and 2 (purple) and nonhotspot input 3 (blue). The right side of Figure 6Aiv shows the summed temporal STA from all inputs in the center region (black), the hotspots (red), and the surround region including hotspots (gray). The similarity between the gray and red traces qualitatively indicates that this cell's hotspots contained the majority of the signal in the STA's surround.

To quantify the amount of surround signal carried by the hotspots, we applied a linear regression between the average STA in the hotspots and the surround (including hotspots). We illustrate this process for three GCs with diverse spatial organization in Figure 6B. The left column shows the spatial distribution of their hotspots, the middle column shows their summed temporal STAs, and the right column compares the signal in the hotspots and overall surround. The right column of Figure 6B shows the linear regression (black line) of the hotspot (*y*‐axis) and overall surround (*x*‐axis) STAs from the middle column. Each red dot in the scatter plot represents the relationship at a different time point and the slope of the black line (*β*, black number) is an estimate of the percent signal in the overall surround contained in the hotspots. For example, the β of the bottom cell is 0.712, implying that hotspots contained 71.2% of the signal strength in the overall surround. As this process involves a circular relationship (the data are used to fit the model, the model is used to determine hotspots, and the β compares the hotspots to the data) we utilized cross‐validation to separate the hotspot identification and β calculation steps. Specifically, hotspots were identified using a subset of the data and determination of hotspot efficacy was determined using a different subset of the data.

The red dots in Figure 6C show the relationship between the percent of hotspot inputs in the surround (*x*‐axis) and *β* (*y*‐axis) for a population of GCs that had a model fit with an antagonistic surround (cells with surround subfilter 1, *N* = 345). Hotspots were on average 11.5% of the inputs in the surround, and had an average beta of 24%. The ordering method used to identify hotspots (red trace) was validated by comparing to an alternative method: ordering the surround inputs by ascending distance from the center (blue trace). The two methods were comparable, with the hotspots outperforming by up to 5% initially. However, the hotspots achieved their performance while selecting inputs that were, on average, quite distant from the center (Fig. 6D, red trace) compared to the distance ordering method (blue trace). The hotspot approach is therefore a reasonable alternative to identifying the spatial structure of the surround which does not rely on the assumption of Gaussian symmetry. Furthermore, because the cross‐validation technique applied to choose and evaluate the hotspots required splitting the data the actual performance of the hotspots is likely underestimated. All hotspots presented henceforth were identified using the full body of data rather than the subset for cross‐validation.

### Hotspots are asymmetrically distributed

GCs can receive asymmetrical synaptic inputs which confer specific functionality (Fried et al. 2002; Briggman et al. 2011), but this asymmetry has not been described in the surround region of linear receptive field maps. We therefore studied the spatial arrangement of hotspots in 256 cells (cells whose RF went off‐screen were manually removed) to determine whether they have symmetrical surrounds. We aligned GCs relative to their major and minor axes from the model fit in order to provide an internal frame of reference for the distribution of inputs. The top left part of Figure 6E shows how the cell's inputs were rotated (white curved arrow) around the center so that the major and minor axes were aligned with the *x* and *y* axes, respectively, and then normalized into standard deviation space (Fig. 6E, top right). The hotspots for the example cell were asymmetrically organized and their center of mass (black line and dot) was nearly 4 SD from the receptive field center.

We performed a similar analysis for the full GC population and found a diverse distribution of symmetries (Fig. 6E, bottom). Each colored dot represents the center of mass for one cell, as exemplified by the black dot and line which is carried from the top right part of Figure 6E. Because GCs from multiple retinas were combined we could not distinguish a center of mass in the top (0–180 degrees) from the bottom (180–360 degrees) for each cell. Data points in the lower half of the polar plot were rotated 180 degrees. A total of 127 of 256 GCs were classified as asymmetrical (red dots) because their receptive field surround had a center of mass which differed statistically from the origin (Wilcoxon signed‐rank test, *P* < 0.01). The hotspots of the remaining 129 GCs were not statistically asymmetrical (green dots) and had an average center of mass that was closer to the origin. Surround asymmetry did not show a significant preference for the major or minor axis of the receptive field center (*χ* ^2^ test, *P* >= 0.05). We conclude that the surround is frequently non‐Gaussian due to both local (heterogeneity) and global (asymmetry) irregularities in its microstructure.

## Discussion

In this report we demonstrate that mouse GCs have an antagonistic surround which has not been previously described in isolated linear receptive fields maps (Kerschensteiner et al. 2008; Koehler et al. 2011; Della Santina et al. 2013). Based on our systematic study of GC STAs, we show that this center–surround receptive field exhibits space–time inseparability suggesting that previously proposed separable models (Chichilnisky and Kalmar 2002) may mischaracterize receptive field components. Most interestingly, we demonstrate that space–time inseparability in the center–surround receptive field can be accounted for by a model consisting of a summed set of separable subfilters (the SoSS model). The spatial and temporal properties of these subfilters differ between ON‐ and OFF‐dominated GCs, suggesting they reflect distinguishing underlying features of different GC types. Moreover, contrary to the common assumption of a radially homogeneous receptive field surround around the center, we find that GC surrounds contain hotspot inputs that are nonuniformly distributed. These findings improve the connection between spatial and temporal filtering of mammalian GCs and shed new light on the rules that govern their linear receptive field organization.

### The temporal tuning of most GCs is more complicated than a single band‐pass filter

Consistent with previously reported temporal frequencies from mouse behavior (0.04–12 Hz) (Umino et al. 2008) and GCs (0.15–10 Hz) (Pandarinath et al. 2010; Wang et al. 2011), the average tuning of our subfilter types ranged from 0.8 to 5 Hz. The temporal filtering of individual GCs was found to have a single band‐pass characteristic (Chichilnisky 2001; Pandarinath et al. 2010; Wang et al. 2011). In contrast, we observed three subfilters (types 1, 2, and 3) in the receptive field center of the majority of GCs (83%) which would function as a dual band‐pass filter. We did observe a single band‐pass filter in the surround of some GCs, where only subfilter types 4 and 5 interact. In the absence of spatial nonlinearities, these results predict temporal tuning to spatially uniform stimuli should have up to six band‐pass peaks due to the possible pair‐wise interactions between the two centers and three antagonistic polarity subfilter types.

### GC linear center–surround receptive fields are space–time inseparable

Past studies using a difference‐of‐Gaussians (DoG) model concluded that an antagonistic surround was weak or not detectable in the linear receptive field maps of mouse GCs. To the contrary, when we applied more general approaches we identified an antagonistic surround in a majority of cells. This success implies that some of the assumptions underlying the DoG model are not valid. For example, the comparisons of a single Gaussian to a DoG spatial profile would be skewed away from the DoG when the surround's signal is focused in a small number of hotspots (although it would be preferred at sufficiently low signal‐to‐noise ratios). These assumptions continue to be adopted in the context of white noise checkerboard mapping because of the lack of direct contradictory evidence and an absence of good alternatives.

In light of this, the work presented here makes two contributions to our understanding of inseparability in the RF. First, we utilized spatial averaging to smooth non‐Gaussian imperfections and identified space–time inseparability in linear receptive fields mapped by white noise checkerboards. This corroborates past observations of space–time inseparability made using classical stimuli (e.g., spots and annuli), and confirms separability cannot be generally assumed (Derrington and Lennie 1982; Enroth‐Cugell et al. 1983; Dawis et al. 1984; Frishman et al. 1987). Second, we identified that mixing space–time separable subfilters with distinct spatiotemporal tuning (the SoSS model) accounts for a significant portion of this inseparability. Of the many ways that inseparability could have manifested (i.e., nonlinear dependence of temporal filter parameters on space), this is arguably the simplest. The SoSS model therefore maintains the analytical tractability of past approaches while improving on their accuracy and general applicability.

### Spatial distribution of the surround

Our observation that the antagonistic surround preferentially localizes in hotspots that are organized inhomogenously extends recent work in the receptive field center. Specifically, GCs’ center was found to deviate from a Gaussian profile in high‐resolution receptive field maps due to the microstructure of its dendritic tree and inhomogeneity of bipolar cell inputs (Schwartz et al. 2012). Because the surround extends far beyond the GC's dendritic field (Volgyi et al. 2009; Pang and Wu 2011), its inhomogeneity likely reflects the connectivity patterns and dendritic microstructure of upstream amacrine and horizontal cells. Identifying such local irregularities will likely play an important role in explaining the spatial information processing of the surround, as has been found in the RF center (Soo et al. 2011). In addition, the asymmetrical organization of hotspots may be the functional manifestation of asymmetric inputs onto GCs that may underlie their direction selectivity (Fried et al. 2002; Briggman et al. 2011). Further experiments are needed to evaluate these contributions.

### Potential synaptic origins of subfilter types

Subtypes within the ganglion cell population are believed to encode different features in ~30 parallel channels (Roska and Werblin 2001; Baden et al. 2016). The nature of these feature channels is an area of active study, but it is clear that the linear receptive fields we measure are incomplete representations of these feature channels. Given that distinct synaptic circuits likely underlie all ~30 subtypes’ feature processing, it is interesting that we observe five subfilters with such distinct responses. We found little evidence to suggest that the subfilters had noteworthy subtyping potential, and therefore surmise that they reflect the contributions of upstream synaptic circuits common to many subtypes.

The distinct spatiotemporal properties and polarities of the subfilter types suggest their possible synaptic origins. For example, our center subfilter 1 is consistent with ON and OFF bipolar cell inputs driven directly by photoreceptors because of its high strength and spatial profile/polarity that match the receptive field center (Famiglietti and Kolb 1976). The narrow spatial profiles and antagonistic polarity of centers 2 and 3 are consistent with polysynaptic pathways comprised of narrow‐field horizontal or amacrine cells (Werblin et al. 1988). Surround 1 had the broad antagonistic spatial profile associated with the classic surround, consistent with the involvement of polysynaptic pathways involving wide‐field horizontal or amacrine cells (Cook and McReynolds 1998). Despite its spatial breadth and additional synapses, surround 1 had fast temporal tuning indicating the involvement of active signal shaping (Bloomfield 1996; Sivyer and Williams 2013). In contrast, surround 2 had a smaller profile and center polarity, suggesting that the two surround components are driven by different polysynaptic pathways. Both center 3 and surround 2 were temporally slow, possibly because they had some synaptic mechanisms in common.

### Comparison to previous measures and models of center–surround receptive fields

Although recent investigations have identified nonlinear interactions in the center and surround, here we characterized only the linear interactions, which are a major component of the receptive field at the target resolution (Bolinger and Gollisch 2012; Takeshita and Gollisch 2014). Models were compared based on adjusted measures of squared errors similar to those applied in past studies (Park and Pillow 2011), but no claims are made regarding firing rate prediction. Some features of our model were presaged by the modified difference‐of‐Gaussians model which was proposed to account for the spatiotemporal dependence of cat *X*‐cell responses (Enroth‐Cugell et al. 1983; Dawis et al. 1984). Like our SoSS model, it allows each spatial Gaussian to be associated with different temporal properties. However, where we define a full spatiotemporal filter, their model does not integrate a specific temporal filter.

Our findings also validate other evidence from these studies. First, the finding of space–time inseparability within GC surrounds but not centers (Dawis et al. 1984) is supported by the common spatial profiles of our center subfilter types (1–3) but different profiles in the surround (types 4–5). Second, the receptive field surround of cat X GCs was observed to have higher frequency tuning than the center (Frishman et al. 1987), consistent with the high tuning of our antagonistic surround (surround 1) compared to the classical center (center 1). Finally, the hotspot asymmetry we observe in Figure 6 supports evidence for asymmetry in the surround of retinal X GCs (Dawis et al. 1984).

## Conflict of Interest

No conflicts to report for any authors.
